# Residual Lung Abnormalities in Survivors of Severe or Critical COVID-19 at One-Year Follow-Up Computed Tomography: A Narrative Review Comparing the European and East Asian Experiences

**DOI:** 10.3390/tomography10010003

**Published:** 2023-12-30

**Authors:** Andrea Borghesi, Pietro Ciolli, Elisabetta Antonelli, Alessandro Monti, Alessandra Scrimieri, Marco Ravanelli, Roberto Maroldi, Davide Farina

**Affiliations:** Department of Medical and Surgical Specialties, Radiological Sciences and Public Health, University of Brescia, ASST Spedali Civili of Brescia, Piazzale Spedali Civili, 1, I-25123 Brescia, Italy; p.ciolli@unibs.it (P.C.); e.antonelli004@unibs.it (E.A.); a.monti006@unibs.it (A.M.); ascrimieri@sirm.org (A.S.); marco.ravanelli@unibs.it (M.R.); roberto.maroldi@unibs.it (R.M.); davide.farina@unibs.it (D.F.)

**Keywords:** SARS-CoV-2, COVID-19, multidetector computed tomography, follow-up studies

## Abstract

The literature reports that there was a significant difference in the medical impact of the coronavirus disease (COVID-19) pandemic between European and East Asian countries; specifically, the mortality rate of COVID-19 in Europe was significantly higher than that in East Asia. Considering such a difference, our narrative review aimed to compare the prevalence and characteristics of residual lung abnormalities at one-year follow-up computed tomography (CT) after severe or critical COVID-19 in survivors of European and East Asian countries. A literature search was performed to identify articles focusing on the prevalence and characteristics of CT lung abnormalities in survivors of severe or critical COVID-19. Database analysis identified 16 research articles, 9 from Europe and 7 from East Asia (all from China). Our analysis found a higher prevalence of CT lung abnormalities in European than in Chinese studies (82% vs. 52%). While the most prevalent lung abnormalities in Chinese studies were ground-glass opacities (35%), the most prevalent lung abnormalities in European studies were linear (59%) and reticular opacities (55%), followed by bronchiectasis (46%). Although our findings required confirmation, the higher prevalence and severity of lung abnormalities in European than in Chinese survivors of COVID-19 may reflect a greater architectural distortion due to a more severe lung damage.

## 1. Introduction

Almost four years have elapsed since the first outbreak of the coronavirus disease (COVID-19) that occurred in Wuhan at the end of 2019 [1]. Although a few sporadic cases of COVID-19 were registered in Europe at the end of December 2019, the first European COVID-19 outbreak was identified on 21 February 2020, in Northern Italy [2,3]. After this first cluster of COVID-19, the severe acute respiratory syndrome coronavirus 2 (SARS-CoV-2) infection quickly spread throughout Europe. On 3 October 2023, the World Health Organization (WHO) reported a number of confirmed cases of SARS-CoV-2 infection totaling 277,468,232 in Europe with 2,253,849 deaths, resulting in an overall mortality rate of 0.8% [4].

Fortunately, following the massive introduction of COVID-19 vaccination programs in European countries—on 1 October 2023, the total number of vaccine doses administered in Europe was 1,736,552,311 [4]—the severity of the disease has significantly decreased, and the situation is gradually returning to normalcy [5,6,7,8]. Specifically, the WHO declared on 5 May 2023 that the global emergency caused by SARS-CoV-2 was over—and we hope it will be forever.

Unfortunately, not all survivors of COVID-19 have recovered their previous health status after the SARS-CoV-2 infection, with some of them, especially those who presented severe or critical disease, still complaining of symptoms related to the disease and presenting with lung abnormalities on chest computed tomography (CT) several months after recovery [9,10,11,12,13,14,15,16,17,18,19].

To date, the number of papers reporting the long-term sequelae of COVID-19 one year or more after infection is progressively increasing, particularly those related to residual lung abnormalities observed on follow-up chest CT [11,12,13,14,15,16,17,18,19,20,21,22,23,24,25,26,27,28,29,30,31,32,33,34,35,36,37,38,39,40,41,42,43,44,45,46,47,48,49]. The frequency of lung parenchymal abnormalities on chest CT examinations performed at one-year follow-up varies according to the severity of COVID-19 (it is greater in patients with severe or critical disease), age (it is greater in patients aged 50 years or older), sex (it is greater in men), length of hospitalization (it is greater in longer hospitalization lengths), invasive ventilation use (it is greater in patients who required invasive ventilation), and sample selection [11,12,13,14,15,16,17,18,19,20,21,22,23,24,25,26,27,28,29,30,31,32,33,34,35,36,37,38,39,40,41,42,43,44,45,46,47,48,49].

Based on the literature, residual lung abnormalities on chest CT are relatively common in survivors of COVID-19 [11,12,13,14,15,16,17,18,19,20,21,22,23,24,25,26,27,28,29,30,31,32,33,34,35,36,37,38,39,40,41,42,43,44,45,46,47,48,49]. At one-year follow-up chest CT, survivors with the lowest frequency of lung parenchymal abnormalities were those with previous mild to moderate disease [14,20,21,24,25,26,29,32,33,35,43,44,45,46,47,48,49]. These patients have mild to moderate symptoms with a blood oxygen saturation ≥ 90% and without signs of severe pneumonia [50].

In contrast, survivors of COVID-19 with previous severe or critical disease presented the highest frequency of lung parenchymal abnormalities at one-year follow-up chest CT [14,20,21,24,25,26,29,32,33,34,35,43,44,45,46,47,48,49]. Patients with severe to critical COVID-19 have a blood oxygen saturation < 90%, signs of severe pneumonia and severe respiratory distress [50]. These patients need to be hospitalized and, depending on the disease severity, should receive immediate respiratory support (high-flow nasal oxygen, non-invasive ventilation, or invasive mechanical ventilation) [33,50].

Knowledge of the frequency and CT characteristics of residual lung abnormalities in survivors of COVID-19 is of paramount importance, as it can aid radiologists and clinicians in differentiating long-term post-COVID-19 sequelae from other interstitial lung diseases, preventing future misdiagnoses.

Currently, several systematic reviews and meta-analyses on long-term CT lung abnormalities in survivors of COVID-19 have been published [18,26,31,47,48,49]. However, to the best of our knowledge, no literature review has yet compared the pulmonary sequelae in survivors after severe or critical COVID-19 at one-year follow-up CT in the European and East Asian populations.

In a previous study, Yamamoto and Bauer [51] reported a significant difference in the medical impact of the COVID-19 pandemic between European countries (such as Spain, Italy, United Kingdom, France, and Germany) and East Asian countries (such as China, Japan, South Korea, and Taiwan). In particular, the authors found that the mortality rate of COVID-19 in Europe was significantly higher than that in East Asia [51].

Considering such a difference, it is plausible to assume that the frequency and severity of the residual lung abnormalities observed on follow-up chest CT in the European population are greater than those observed in the East Asian population. Therefore, our narrative review aimed to compare the frequency and CT characteristics of residual lung abnormalities at one-year follow-up in patients of European and East Asian countries by focusing the analysis on survivors of severe or critical COVID-19.

## 2. Materials and Methods

### 2.1. Literature Search Strategy

A literature search of the PubMed/MEDLINE, Scopus, and Web of Science databases was performed to identify articles focusing on the frequency and CT characteristics of long-term CT lung abnormalities in survivors of COVID-19. Different combinations of the following terms were used in the search: COVID-19; SARS-CoV-2; chest CT; CT; one year/one-year; 1 year/1-year; long term; and follow-up. The final search of the three databases was conducted on 29 September 2023.

### 2.2. Study Selection Criteria

For this literature search, the following inclusion criteria were considered: (a) articles written in English, (b) articles focused on residual lung abnormalities on CT images in survivors of COVID-19 at one-year follow-up, and (c) studies conducted in European and East Asian countries. Only studies that reported the CT characteristics of residual lung abnormalities and their respective frequencies at one-year follow-up after severe or critical COVID-19 in detail were included. Data regarding patients with mild to moderate disease were not considered in this review as the monitoring strategies for this group of patients differed significantly between countries and healthcare institutions.

Case reports, case series, letters to the editor, editorials, commentaries, conference papers, and review articles were excluded from this review. In addition, articles on long-term residual lung abnormalities that specifically focused on survivors of COVID-19 with underlying comorbidities were excluded.

**Figure 1:**
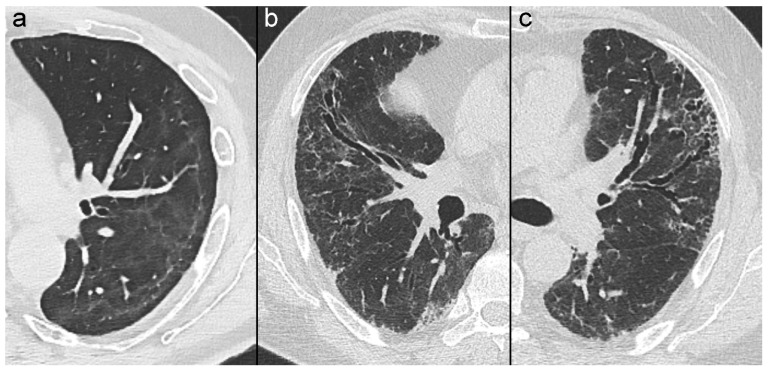
Cropped axial thin-section computed tomography (CT) images with lung window setting show some examples of long-term chest CT findings in survivors of coronavirus disease (COVID-19): (**a**) ground-glass and subpleural curvilinear opacities; (**b**) reticular opacities with architectural distortion and bronchiectasis; (**c**) reticular opacities with architectural distortion and traction bronchiectasis.

The CT findings considered for this review included the following lung abnormalities: ground-glass opacities; reticular opacities; linear opacities; consolidations; bronchiectasis; traction bronchiectasis; and honeycombing (Figure 1).

Using the term reticular opacities, we also included the following chest CT findings: reticulations, reticular abnormalities, reticular lesions, and reticular patterns. Using the term linear opacities, we also included the following chest CT findings: bands; curvilinear bands; interlobular thickening; interlobular septal thickening; irregular lines; lines; parenchymal bands; subpleural curvilinear opacities; and subpleural lines. In the case where more than one of these linear opacities were reported, only the one with the highest prevalence was considered.

The three databases were searched by an experienced thoracic radiologist (A.B.) assisted by two radiology residents (P.C. and E.A.) with three and four years of experience in CT imaging, respectively.

### 2.3. Data Extraction

For each included article, we collected the following data: (a) article details (first author, month/year of submission/publication, country of origin, and design); (b) study sample characteristics (number of patients, age, number/percentage of men, and number/percentage of survivors of severe or critical COVID-19); and (c) prevalence, CT characteristics, and extent of residual lung abnormalities at one-year follow-up grouped by European and East Asian countries.

## 3. Results

Based on the predefined selection criteria, 16 original research articles were included in this review (9 from Europe and 7 from East Asia).

The main characteristics of the included articles are listed in Table 1 and Table 2.

The European articles were published in the following countries: Italy, four; Spain, two; France, one; Belgium, one; and the Netherlands, one. Conversely, all articles selected from East Asia were conducted in China. The submission dates of the included studies ranged from April 2021 to September 2022.

As shown in Table 1 and Table 2, all articles except one presented data from a prospective analysis. Only one study, conducted in Europe (specifically, in one of the hot spots of the pandemic in Northern Italy), presented data from a retrospective analysis [28].

Among the 16 selected articles, 7 (43.8%) were multicenter (5 from Europe and 2 from China) [11,20,29,33,34,35,39]. The selected studies included a total of 3683 patients after COVID-19 infection (1515 patients from Europe and 2168 patients from China). The mean or median age of the survivors of COVID-19 reported in these articles ranged from 39 to 68 years (59 to 68 years in European studies and 39 to 60 years in Chinese studies). The overall percentage of men was 56%, ranging from 19 to 79% (55 to 79% in European studies and 19 to 57% in Chinese studies). However, considering only severe or critical COVID-19 and excluding patients from the study of Liao et al. (performed on health care workers) [44], the mean/median age of the Chinese patients ranged from 51 to 60 years with a percentage of men ranging from 50 to 67%.

As reported in Table 1 and Table 2, 7/16 (43.8%) studies (5 from Europe and 2 from China) included only survivors of COVID-19 after severe or critical infection, whereas the remaining 9/16 (56.2%) studies included survivors of COVID-19 after both mild to moderate and severe to critical infections.

Overall, the selected studies comprised a total of 1383 survivors of COVID-19 after severe or critical infection (843 patients from Europe and 540 patients from China). Considering only such a group of patients, although the mean or median age of survivors of COVID-19 was similar, the overall percentage of men increased from 56% to 62%.

Among the survivors of COVID-19 after severe or critical infection, 923/1383 (66.7%) had a one-year chest CT follow-up (477 patients from European countries and 446 patients from China). Only the residual lung abnormalities on CT images from this group of 923 patients were included in our analyses (Table 3 and Table 4).

Among the selected studies, 10/16 (62.5%) articles (6 from Europe and 4 from China) clearly stated that CT image analyses had been performed by at least one experienced radiologist [11,20,21,25,28,29,33,36,38,42], with 6 (60%) articles (4 from Europe and 2 from China) including a radiologist who was an expert in thoracic imaging [11,25,29,36,38,42], whereas 2/16 (12.5%) articles stated that the CT images had been analyzed by a radiologist without specifying whether the radiologist was an expert [35,45]. The remaining four (25%) articles (two from Europe and two from China) did not specify who analyzed the CT images [34,39,43,44].

As shown in Table 3, the reported prevalence of any CT lung abnormalities at one-year follow-up ranged from 64% to 100% in the European studies. Among the European articles, only one study (prospective multicenter) from Italy did not report the overall prevalence of any CT lung abnormalities [39]. A total of 362/440 (82%) European patients had residual lung abnormalities on CT images performed one year after severe or critical COVID-19.

As shown in Table 4, the reported prevalence of any CT lung abnormalities at one-year follow-up ranged from 24% to 87% in the Chinese articles. Among the Chinese articles, only one study (prospective single-center) did not report the overall prevalence of any CT lung abnormalities [45]. A total of 210/403 (52%) Chinese patients had residual lung abnormalities on CT images performed one year after severe or critical COVID-19. Excluding the study of Liao et al. [44], the percentage of Chinese patients with residual lung abnormalities was 60%.

Among the selected European studies, in 4/9 (44%) articles, the most prevalent CT findings were reticular opacities [28,35,36,42]; in 4/9 (44%) articles, the most prevalent CT findings were linear opacities [39]; and in the remaining article, the most prevalent CT finding was ground-glass opacities [29] (Table 3).

**Figure 2:**
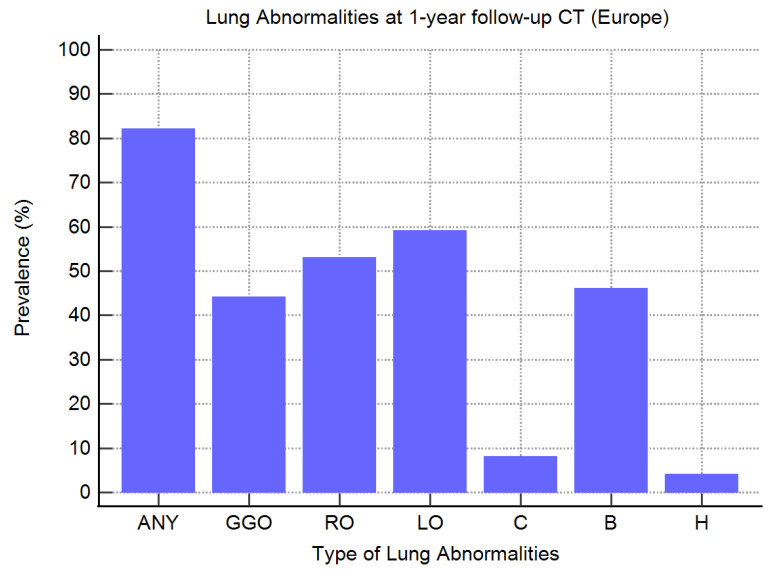
Prevalence of different types of CT lung abnormalities in survivors of severe or critical COVID-19 at one-year follow-up (European studies). ANY, any residual lung abnormalities; GGO, ground-glass opacities; RO, reticular opacities; LO, linear opacities; C, consolidation; B, bronchiectasis; H, honeycombing.

Considering all European articles, the most frequent residual lung abnormalities at one-year follow-up CT were reticular opacities, identified in 254/477 (53%) survivors of COVID-19, followed by bronchiectasis (with or without traction), identified in 219/477 (46%) patients, and ground-glass opacities, identified in 212/477 (44%) patients (Table 3 and Figure 2). As shown in Table 3, the prevalence of linear opacities was reported in only 5/9 (56%) studies; in such articles, the prevalence of linear opacities on CT images ranged from 11 to 100%, with an overall prevalence of 59% (123/208 survivors of COVID-19; Table 3 and Figure 2).

Consolidations and honeycombing were the less frequent CT findings at one-year follow-up, with an overall prevalence of 7% (30/413 survivors of COVID-19) and 4% (9/233 survivors of COVID-19), respectively (Table 3 and Figure 2). As shown in Table 3, consolidations were reported in 8/9 (89%) studies; the prevalence of consolidation on CT images was not reported by Eberst et al. [36]. The prevalence of honeycombing was reported in only 4/9 (44%) studies (Table 3). In these articles, the prevalence of honeycombing in the CT images ranged from 1 to 8% [29,36,39,42].

**Figure 3:**
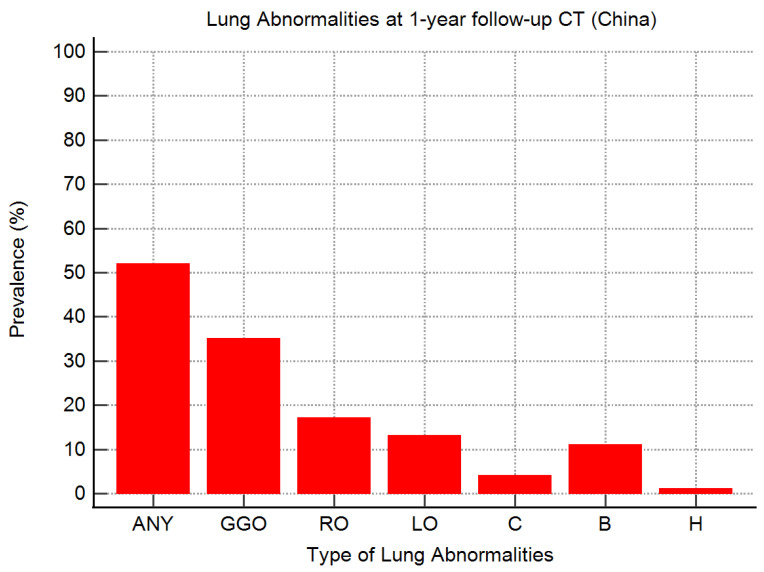
Prevalence of different types of CT lung abnormalities in survivors of severe or critical COVID-19 at one-year follow-up (Chinese studies). ANY, any residual lung abnormalities; GGO, ground-glass opacities; RO, reticular opacities; LO, linear opacities; C, consolidation; B, bronchiectasis; H, honeycombing.

Among the selected Chinese studies, the most prevalent CT findings were ground-glass opacities in 5/7 (71%) articles [21,38,43,44,45], ground-glass opacities together with linear opacities in one article [20], and reticular opacities in the remaining article [11] (Table 4). The most frequent residual lung abnormalities at one-year follow-up CT were ground-glass opacities, identified in 158/446 (35%) survivors of COVID-19 survivors, followed by reticular opacities, identified in 72/432 (17%) patients, liner opacities, identified in 49/384 (13%) survivors of COVID-19, and bronchiectasis (with or without traction), identified in 39/365 (11%) patients (Table 4 and Figure 3).

As shown in Table 4, the prevalence of reticular and linear opacities was reported in 6/7 (86%) studies, reticular opacities were not reported in the study by Zhou et al. [20], and linear opacities were not included in the study by Han et al. [11]. The prevalence of bronchiectasis (including traction bronchiectasis) at one-year follow-up CT was reported in 5/7 (71%) Chinese studies (Table 4); bronchiectasis was not reported in the studies by Zhao et al. [45] and Huang et al. [21].

Consolidation and honeycombing were the less frequent CT findings at one-year follow-up, with an overall prevalence of 4% (17/432 survivors of COVID-19) and 1% (2/289 patients), respectively (Table 4 and Figure 3). As shown in Table 4, the prevalence of consolidation was reported in 6/7 (86%) studies; the prevalence of consolidation on CT images was not reported in the study by Zhou et al. [20]. The prevalence of honeycombing was reported in 3/7 (43%) studies (Table 4); in these articles, the prevalence of honeycombing in the CT images ranged from 0 to 4% [38,43,44].

Excluding the patients from study of Liao et al. [44], the percentage of Chinese patients with ground-glass opacities, reticular opacities, linear opacities, bronchiectasis, consolidation, and honeycombing was 40%, 26%, 19%, 18%, 4%, and 2%, respectively.

Regarding the quantitative assessment of the extent of residual lung abnormalities on CT images, only 5/16 (38%) studies (3 from Europe and 2 from China) estimated the overall percentage of lung involvement: 3 with dedicated software [20,28,44], and 2 with a visual method [25,42] (Table 5). As shown in Table 5, the overall percentage of lung volume affected by residual lung abnormalities on CT images ranged from 0 to 12%, with a higher percentage of lung involvement in European survivors of COVID-19 than in those from China.

In other 4/16 (25%) studies (3 from China and 1 from Europe), the extent of residual lung abnormalities on CT images was evaluated using a semi-quantitative visual method [11,34,43,45]. In the remaining 7/16 (44%) articles, no quantitative or semi-quantitative assessment of the overall extent of lung abnormalities was reported [21,29,33,35,36,38,39].

## 4. Discussion

As reported by Yamamoto and Bauer [51], strong evidence indicates the presence of significant differences in the spread of SARS-CoV-2 infection and COVID-19 severity between European and East Asian countries. Specifically, the authors reported that the mortality rate of COVID-19 in Europe was significantly higher than that in East Asia.

To explain such differences between European and East Asian countries, Yamamoto and Bauer [51] proposed four possible hypothesis, as follows: (a) differences in socio-behavioral aspects and lifestyle between the two regions (e.g., shaking hands, kissing, or hugging one another is an uncommon behavior in East Asian countries); (b) differences in SARS-CoV-2 virulence due to multiple viral infections, with a greater virulence in Europe, probably due to inadequate protection and underestimation of SARS-CoV-2 contagiousness in combination with antibody-dependent enhancement and mutation of the viral RNA genome; (c) differences in individual resistance to SARS-CoV-2 infection (e.g., East Asian populations may have an immune system genetically trained to defend themselves from novel viruses, including coronavirus; and (d) differences in hygiene aspects.

Based on the data reported by Yamamoto and Bauer [51], we considered it plausible to hypothesize that the prevalence and severity of residual lung abnormalities on CT scans after COVID-19 were higher in European than in East Asian patients.

In line with this hypothesis, our review found that the prevalence and severity of residual lung abnormalities on CT images at one-year follow-up after severe or critical COVID-19 were significantly higher in European than in Chinese patients (Table 3 and Table 4). Notably, while the overall prevalence of any CT lung abnormality in European studies was 82%, that in Chinese studies was 52% (60% excluding the study of Liao et al. [44]).

In European studies, the most prevalent lung abnormalities after severe or critical COVID-19 were linear (59%) and reticular opacities (53%), followed by bronchiectasis (46%) and ground-glass opacities (44%) (Figure 2). In contrast, in Chinese studies, the most prevalent lung abnormalities after severe or critical infection were ground-glass opacities (35% or 40% if the study of Liao et al. [44] was excluded). Additionally, the observed prevalence of reticular opacities (17%), linear opacities (13%), and bronchiectasis (11%) in Chinese patients was significantly lower than in European patients (Figure 3). This difference remained even when the patients from the study of Liao et al. [44] were excluded.

The differences between the prevalence and type of residual lung abnormalities among European and Chinese studies likely reflect the different severities of the disease (higher in Europe), as suggested by Yamamoto and Bauer [51].

The residual lung abnormality that mostly reflected the severity and fibrotic evolution of lung damage was bronchiectasis, and its prevalence was significantly higher in European than in Chinese patients (46% in European vs. 11% in Chinese studies). Honeycombing, traditionally considered a CT feature of established pulmonary fibrosis (end-stage pulmonary fibrosis), was more prevalent in European than in Chinese patients (4% in European vs. 1% in Chinese studies). Additionally, we found that the percentage of lung volume affected by residual lung abnormalities on CT images was significantly greater in European than in Chinese patients (Table 5).

In contrast to previous systematic reviews and meta-analyses on this topic [26,31,47,48,49], we found that ground-glass opacities were the most frequently reported residual lung abnormality, yet only in Chinese studies [20,21,38,43,44,45] (Table 2 and Figure 3). In contrast, the most frequently reported residual lung abnormalities in European studies were reticular [28,35,36,42] and linear opacities [25,33,34,39]. Solely in one European study, the most common lung abnormalities were ground-glass opacities [29] (Table 1). Additionally, the overall prevalence of ground-glass opacities in European survivors of COVID-19 was lower than that of linear and reticular opacities, and bronchiectasis.

The differences between our findings and those reported previously [26,31,47,48,49] are probably due to a different method of analyses. Unlike previous systematic reviews [26,31,47,48,49], our study included only chest CT abnormalities in survivors of COVID-19 after severe or critical SARS-CoV-2 infection, and the CT findings were grouped based on the region of origin (Europe vs. China).

The literature also reports that the second most common lung abnormalities in survivors of COVID-19 were fibrotic-like changes [26,31]. In contrast to previous systematic reviews [26,31,47,48], we excluded fibrotic and fibrotic-like changes from our analysis, as this term is rather ambiguous and is affected by the wide variability in its definition across various studies [18,49].

The higher prevalence of linear and reticular opacities, bronchiectasis (including bronchial dilatation and traction bronchiectasis), and honeycombing observed in European than in Chinese patients may reflect a greater architectural lung distortion with a possible evolution to fibrosis due to a more severe lung damage.

Although further studies are required to confirm our data, the major strength of the present review is that it is the first to compare the pulmonary sequelae and their prevalence after severe or critical COVID-19 at one-year follow-up CT in the European and Chinese population.

This study had several limitations. First, it was a narrative (non-systematic) review, and only descriptive statistics were performed. Second, the number of selected articles was relatively small because the inclusion criteria were rather strict, and only papers that reported the CT characteristics of residual lung abnormalities after severe or critical COVID-19 in detail were included. Third, there was heterogeneity in the data of the selected studies; however, to reduce the heterogeneity of the subgroups, only survivors of COVID-19 after severe to critical infection were included. Fourth, there were differences in the age and proportion of men between European and Chinese studies; however, these differences cannot explain the higher prevalence and severity of residual lung abnormalities in Europeans compared to Chinese, and also because all patients were severely or critically ill and the differences in age and proportion of men between European and Chinese survivors were significantly smaller when the patients from the study of Liao et al. (performed on health care workers) [44] were excluded. Fifth, no information regarding underlying comorbidities, smoking history, pulmonary functional tests, or laboratory parameters was assessed, as this review focused only on residual lung abnormalities detected at one-year follow-up CT.

## 5. Conclusions and Future Directions

In conclusion, the prevalence, severity, and extent of residual lung abnormalities at one-year follow-up CT after severe to critical COVID-19 infection were higher in European compared to Chinese patients. In contrast to the Chinese studies, the most frequently reported abnormalities in European articles were linear and reticular opacities, and bronchiectasis, which probably reflect greater architectural lung distortion with possible evolution to fibrosis because of a more severe lung damage.

The findings observed in the present narrative review must be verified in future systematic analyses with a larger number of studies and longer follow-up periods (i.e., beyond one year). However, we believe that our results, together with those of Yamamoto and Bauer [51], are clinically relevant, as they suggest that the European population may be at a greater risk both for death and severe post-infectious sequelae if a pandemic from a new virus were to occur. While hoping that this does not happen, this information could be useful not only for clinicians but also for governments by facilitating the introduction of appropriate measures to prevent infection, and, thereby, reduce deaths and infectious sequelae.

## Figures and Tables

**Table 1:** Study details and patient characteristics in the selected European studies.

First Author	Date †	Country	Design	Study Patient			Severe or Critical COVID-19		
				No.	Age * (Years)	Men	No.	Age * (Years)	Men
Gamberini [39]	September 2021	Italy	Prospective/MC	178	64	129 (72)	178 (100)	64	129 (72)
Eberst [36]	September 2021	France	Prospective/SC	85	68	67 (79)	85 (100)	68	67 (79)
Martino [42]	September 2021	Italy	Prospective/SC	64	68	41 (64)	64 (100)	68	41 (64)
Faverio [29]	October 2021	Italy	Prospective/MC	287	61	213 (74)	90 (31) ^	60	74 (82)
Lorent [35]	January 2022	Belgium	Prospective/MC	299	59	205 (69)	94 (31)	60	78 (83)
Gonzalez [34]	March 2022	Spain	Prospective/MC	181	61	121 (67)	181 (100)	61	121 (67)
Tarraso [33]	May 2022	Spain	Prospective/MC	284	61	157 (55)	52 (18)	63	38 (73)
Van Raaij [25]	August 2022	Netherlands	Prospective/SC	66	61	46 (70)	28 (42)	60	19 (68)
Corsi [28]	September 2022	Italy	Retrospective/SC	71	66	45 (63)	71 (100)	66	45 (63)

Data are presented as number (percentages in round brackets); † Month and year of paper submission; MC, multicenter study; SC; single-center study; * Age is presented as mean or median; ^ patients who have received invasive mechanical ventilation.

**Table 2:** Study details and patient characteristics in the selected East Asian studies (all from China).

First Author	Date †	Country	Design	Study Patient			Severe or Critical COVID-19		
				No.	Age * (Years)	Men	No.	Age * (Years)	Men
Han [11]	April 2021	China	Prospective/MC	62	57	34 (55)	62 (100)	57	34 (55)
Wu [38]	May 2021	China	Prospective/SC	83	60	47 (57)	83 (100)	60	47 (57)
Zhou [20]	May 2021	China	Prospective/MC	120	52	49 (41)	16 (13)	53	8 (50)
Zhao [45]	July 2021	China	Prospective/SC	94	48	54 (57)	43 (46)	51	29 (67)
Huang [21]	August 2021	China	Prospective/SC	1276	59	681 (53)	94 (7)	58	63 (67)
Liao [44]	September 2021	China	Prospective/SC	303	39	59 (19)	190 (63)	39	37 (19)
Li [43]	January 2022	China	Prospective/SC	230	46	116 (50)	52 (23)	55	33 (63)

Data are presented as number (percentages in round brackets); † Month and year of paper submission; MC, multicenter study; SC; single-center study; * Age is presented as mean or median.

**Table 3:** Residual lung abnormalities on chest CT images (European studies).

First Author	Patients with 1-Year CT Follow-Up	CT Lung Abnormalities at 1-Year Follow-Up after Severe or Critical COVID-19						
		Any	GGOs	Reticular Opacities	Linear Opacities	Consolidation	Bronchiectasis (+/− Traction)	Honeycomb
Gamberini [39] °	37 (21)	NA	21 (57)	13 (35)	26 (70)	3 (8)	10 (27)	3 (8)
Eberst [36] *	64 (75)	60 (94)	32 (50)	51 (80)	NA	NA	44 (69)	3 (5)
Martino [42] ^	47 (73)	30 (64)	7 (15)	19 (40)	5 (11)	7 (15)	4 (9)	2 (4)
Faverio [29] *	85 (94)	68 (80)	60 (71)	42 (49)	NA	2 (2)	24 (28)	1 (1)
Lorent [35] †	57 (61)	40 (65)	21 (37)	36 (63)	NA	0 (0)	14 (25)	NA
Gonzalez [34] °	41 (23)	41 (100)	27 (66)	22 (54)	41 (100)	3 (7)	37 (90)	NA
Tarraso [33] *	57 (100)	54 (95)	31 (54)	23 (40)	32 (56)	11 (19)	28 (49)	NA
Van Raaij [25] *	26 (93)	21 (81)	11 (42)	10 (39)	19 (73)	2 (8)	16 (62)	NA
Corsi [28] ^	63 (89)	48 (76)	2 (3)	38 (60)	NA	2 (3)	42 (67)	NA

* CT images analysis performed by at least one thoracic radiologist; ^ CT images analysis performed by at least one experienced radiologist; † CT images analysis performed by at least radiologist; ° Not specified who analyzed the CT images; GGOs, ground-glass opacities; NA, not available.

**Table 4:** Residual lung abnormalities on chest CT images (Chinese studies).

First Author	Patients with 1-Year CT Follow-Up	CT Lung Abnormalities at 1-Year Follow-Up after Severe or Critical COVID-19						
		Any	GGOs	Reticular Opacities	Linear Opacities	Consolidation	Bronchiectasis (+/− Traction)	Honeycomb
Han [11] *	62 (100)	45 (73)	7 (11)	32 (52)	NA	6 (10)	27 (44)	NA
Wu [38] *	83 (100)	20 (24)	19 (23)	3 (4)	5 (6)	0 (0)	1 (1)	0 (0)
Zhou [20] ^	14 (88)	8 (57)	5 (36)	NA	5 (36)	NA	1 (7)	NA
Zhao [45] †	43 (100)	NA	20 (47)	3 (7)	8 (19)	2 (5)	NA	NA
Huang [21] ^	38 (40)	33 (87)	29 (76)	3 (8)	23 (61)	1 (3)	NA	NA
Liao [44] °	158 (83)	63 (40)	43 (27)	2 (1)	5 (3)	7 (4)	2 (1)	0 (0)
Li [43] °	48 (92)	41 (85)	35 (73)	29 (60)	3 (6)	1 (2)	8 (17)	2 (4)

* CT images analysis performed by at least one thoracic radiologist; ^ CT images analysis performed by at least one experienced radiologist; † CT images analysis performed by at least radiologist; ° Not specified who analyzed the CT images; GGOs, ground-glass opacities; NA, not available.

**Table 5:** Extent of CT lung abnormalities in survivors of severe or critical COVID-19.

First Author	Patients with 1-Year CT Follow-Up	Extent of CT Lung Abnormalities at 1-Year Follow-Up	
		Visual Assessment (Percentage)	Software-Based Analysis (Percentage)
Martino [42]	47	5 (0–10)	-
Van Raaij [25]	26	11 (4–26)	-
Corsi [28]	63	-	12 (9–16)
Zhou [20]	14	-	0 (0–0.02)
Liao [44]	158	-	0 (0–0.03)

Data are presented as median (interquartile range in round brackets).

## Data Availability

Not applicable.

## References

[1] Zhu N., Zhang D., Wang W., Li X., Yang B., Song J., Zhao X., Huang B., Shi W., Lu R. (2020). A Novel Coronavirus from Patients with Pneumonia in China, 2019. N. Engl. J. Med..

[2] Cerqua A., Di Stefano R. (2022). When Did Coronavirus Arrive in Europe?. Stat. Methods Appl..

[3] Borghesi A., Maroldi R. (2020). COVID-19 Outbreak in Italy: Experimental Chest X-Ray Scoring System for Quantifying and Monitoring Disease Progression. Radiol. Med..

[4] World Health Organization (WHO) Europe—Coronavirus Disease (COVID-19) Pandemic. https://www.who.int/europe/emergencies/situations/covid-19.

[5] Lopez Bernal J., Andrews N., Gower C., Robertson C., Stowe J., Tessier E., Simmons R., Cottrell S., Roberts R., O’Doherty M. (2021). Effectiveness of the Pfizer-BioNTech and Oxford-AstraZeneca Vaccines on Covid-19 Related Symptoms, Hospital Admissions, and Mortality in Older Adults in England: Test Negative Case-Control Study. BMJ.

[6] Lee J.E., Hwang M., Kim Y.-H., Chung M.J., Sim B.H., Chae K.J., Yoo J.Y., Jeong Y.J. (2022). Imaging and Clinical Features of COVID-19 Breakthrough Infections: A Multicenter Study. Radiology.

[7] Borghesi A., Maroldi R. (2022). Vaccination and Reduced Severity of COVID-19 Pneumonia Viewed at Chest Radiography. Radiology.

[8] Graña C., Ghosn L., Evrenoglou T., Jarde A., Minozzi S., Bergman H., Buckley B.S., Probyn K., Villanueva G., Henschke N. (2022). Efficacy and Safety of COVID-19 Vaccines. Cochrane Database Syst. Rev..

[9] So M., Kabata H., Fukunaga K., Takagi H., Kuno T. (2021). Radiological and Functional Lung Sequelae of COVID-19: A Systematic Review and Meta-Analysis. BMC Pulm. Med..

[10] Besutti G., Monelli F., Schirò S., Milone F., Ottone M., Spaggiari L., Facciolongo N., Salvarani C., Croci S., Pattacini P. (2022). Follow-Up CT Patterns of Residual Lung Abnormalities in Severe COVID-19 Pneumonia Survivors: A Multicenter Retrospective Study. Tomography.

[11] Han X., Fan Y., Alwalid O., Zhang X., Jia X., Zheng Y., Shi H. (2021). Fibrotic Interstitial Lung Abnormalities at 1-Year Follow-up CT after Severe COVID-19. Radiology.

[12] Pan F., Yang L., Liang B., Ye T., Li L., Li L., Liu D., Wang J., Hesketh R.L., Zheng C. (2022). Chest CT Patterns from Diagnosis to 1 Year of Follow-up in Patients with COVID-19. Radiology.

[13] Lee K.S., Wi Y.M. (2022). Residual Lung Lesions at 1-Year CT after COVID-19. Radiology.

[14] Luger A.K., Sonnweber T., Gruber L., Schwabl C., Cima K., Tymoszuk P., Gerstner A.K., Pizzini A., Sahanic S., Boehm A. (2022). Chest CT of Lung Injury 1 Year after COVID-19 Pneumonia: The CovILD Study. Radiology.

[15] Marando M., Fusi-Schmidhauser T., Tamburello A., Grazioli Gauthier L., Rigamonti E., Argentieri G., Puligheddu C., Pagnamenta A., Valenti A., Pons M. (2022). 1-Year Radiological, Functional and Quality-of-Life Outcomes in Patients with SARS-CoV-2 Pneumonia—A Prospective Observational Study. NPJ Prim. Care Respir. Med..

[16] Zangrillo A., Belletti A., Palumbo D., Calvi M.R., Guzzo F., Fominskiy E.V., Ortalda A., Nardelli P., Ripa M., Baiardo Redaelli M. (2022). One-Year Multidisciplinary Follow-Up of Patients With COVID-19 Requiring Invasive Mechanical Ventilation. J. Cardiothorac. Vasc. Anesth..

[17] Ribeiro Carvalho C.R., Lamas C.A., Chate R.C., Salge J.M., Sawamura M.V.Y., De Albuquerque A.L.P., Toufen Junior C., Lima D.M., Garcia M.L., Scudeller P.G. (2023). Long-Term Respiratory Follow-up of ICU Hospitalized COVID-19 Patients: Prospective Cohort Study. PLoS ONE.

[18] Kanne J.P., Little B.P., Schulte J.J., Haramati A., Haramati L.B. (2023). Long-Term Lung Abnormalities Associated with COVID-19 Pneumonia. Radiology.

[19] Guo Y., Wang H., Xiao M., Guan X., Lei Y., Diao T., Long P., Zeng R., Lai X., Cai H. (2023). Long-Term Outcomes of COVID-19 Convalescents: An 18.5-Month Longitudinal Study in Wuhan. Int. J. Infect. Dis..

[20] Zhou F., Tao M., Shang L., Liu Y., Pan G., Jin Y., Wang L., Hu S., Li J., Zhang M. (2021). Assessment of Sequelae of COVID-19 Nearly 1 Year After Diagnosis. Front. Med..

[21] Huang L., Yao Q., Gu X., Wang Q., Ren L., Wang Y., Hu P., Guo L., Liu M., Xu J. (2021). 1-Year Outcomes in Hospital Survivors with COVID-19: A Longitudinal Cohort Study. Lancet.

[22] Huang L., Li X., Gu X., Zhang H., Ren L., Guo L., Liu M., Wang Y., Cui D., Wang Y. (2022). Health Outcomes in People 2 Years after Surviving Hospitalisation with COVID-19: A Longitudinal Cohort Study. Lancet Respir. Med..

[23] Vijayakumar B., Tonkin J., Devaraj A., Philip K.E.J., Orton C.M., Desai S.R., Shah P.L. (2022). CT Lung Abnormalities after COVID-19 at 3 Months and 1 Year after Hospital Discharge. Radiology.

[24] Bocchino M., Lieto R., Romano F., Sica G., Bocchini G., Muto E., Capitelli L., Sequino D., Valente T., Fiorentino G. (2022). Chest CT–Based Assessment of 1-Year Outcomes after Moderate COVID-19 Pneumonia. Radiology.

[25] Van Raaij B.F.M., Stöger J.L., Hinnen C., Penfornis K.M., De Jong C.M.M., Klok F.A., Roukens A.H.E., Veldhuijzen D.S., Arbous M.S., Noordam R. (2022). Fibrotic-like Abnormalities Notably Prevalent One Year after Hospitalization with COVID-19. Respir. Med. Res..

[26] Watanabe A., So M., Iwagami M., Fukunaga K., Takagi H., Kabata H., Kuno T. (2022). One-year Follow-up CT Findings in COVID -19 Patients: A Systematic Review and Meta-analysis. Respirology.

[27] Bellan M., Baricich A., Patrucco F., Zeppegno P., Gramaglia C., Balbo P.E., Carriero A., Amico C.S., Avanzi G.C., Barini M. (2021). Long-Term Sequelae Are Highly Prevalent One Year after Hospitalization for Severe COVID-19. Sci. Rep..

[28] Corsi A., Caroli A., Bonaffini P.A., Conti C., Arrigoni A., Mercanzin E., Imeri G., Anelli M., Balbi M., Pace M. (2022). Structural and Functional Pulmonary Assessment in Severe COVID-19 Survivors at 12 Months after Discharge. Tomography.

[29] Faverio P., Luppi F., Rebora P., D’Andrea G., Stainer A., Busnelli S., Catalano M., Modafferi G., Franco G., Monzani A. (2022). One-Year Pulmonary Impairment after Severe COVID-19: A Prospective, Multicenter Follow-up Study. Respir. Res..

[30] Chen Y., Ding C., Yu L., Guo W., Feng X., Yu L., Su J., Xu T., Ren C., Shi D. (2021). One-Year Follow-up of Chest CT Findings in Patients after SARS-CoV-2 Infection. BMC Med..

[31] Lee J.H., Yim J.-J., Park J. (2022). Pulmonary Function and Chest Computed Tomography Abnormalities 6–12 Months after Recovery from COVID-19: A Systematic Review and Meta-Analysis. Respir. Res..

[32] Lenoir A., Christe A., Ebner L., Beigelman-Aubry C., Bridevaux P.-O., Brutsche M., Clarenbach C., Erkosar B., Garzoni C., Geiser T. (2023). Pulmonary Recovery 12 Months after Non-Severe and Severe COVID-19: The Prospective Swiss COVID-19 Lung Study. Respiration.

[33] Tarraso J., Safont B., Carbonell-Asins J.A., Fernandez-Fabrellas E., Sancho-Chust J.N., Naval E., Amat B., Herrera S., Ros J.A., Soler-Cataluña J.J. (2022). Lung Function and Radiological Findings 1 Year after COVID-19: A Prospective Follow-Up. Respir. Res..

[34] González J., Zuil M., Benítez I.D., De Gonzalo-Calvo D., Aguilar M., Santisteve S., Vaca R., Minguez O., Seck F., Torres G. (2022). One Year Overview and Follow-Up in a Post-COVID Consultation of Critically Ill Patients. Front. Med..

[35] Lorent N., Vande Weygaerde Y., Claeys E., Guler Caamano Fajardo I., De Vos N., De Wever W., Salhi B., Gyselinck I., Bosteels C., Lambrecht B.N. (2022). Prospective Longitudinal Evaluation of Hospitalised COVID-19 Survivors 3 and 12 Months after Discharge. ERJ Open Res..

[36] Eberst G., Claudé F., Laurent L., Meurisse A., Roux-Claudé P., Barnig C., Vernerey D., Paget-Bailly S., Bouiller K., Chirouze C. (2022). Result of One-Year, Prospective Follow-up of Intensive Care Unit Survivors after SARS-CoV-2 Pneumonia. Ann. Intensive Care.

[37] Zhao Y., Wang D., Mei N., Yin B., Li X., Zheng Y., Xiao A., Yu X., Qiu X., Lu Y. (2021). Longitudinal Radiological Findings in Patients With COVID-19 With Different Severities: From Onset to Long-Term Follow-Up After Discharge. Front. Med..

[38] Wu X., Liu X., Zhou Y., Yu H., Li R., Zhan Q., Ni F., Fang S., Lu Y., Ding X. (2021). 3-Month, 6-Month, 9-Month, and 12-Month Respiratory Outcomes in Patients Following COVID-19-Related Hospitalisation: A Prospective Study. Lancet Respir. Med..

[39] Gamberini L., Mazzoli C.A., Prediletto I., Sintonen H., Scaramuzzo G., Allegri D., Colombo D., Tonetti T., Zani G., Capozzi C. (2021). Health-Related Quality of Life Profiles, Trajectories, Persistent Symptoms and Pulmonary Function One Year after ICU Discharge in Invasively Ventilated COVID-19 Patients, a Prospective Follow-up Study. Respir. Med..

[40] Li Y., Han X., Huang J., Alwalid O., Jia X., Yuan M., Cao Y., Shao G., Cui Y., Liu J. (2021). Follow-up Study of Pulmonary Sequelae in Discharged COVID-19 Patients with Diabetes or Secondary Hyperglycemia. Eur. J. Radiol..

[41] Bernardinello N., Cocconcelli E., Giraudo C., Daverio M., Castelli G., Petrarulo S., Bovo M., Fichera G., Cavinato S., Cattelan A.M. (2023). Predictors of Pulmonary Sequelae after COVID-19 Pneumonia: A 12-Month Follow-up Study. Front. Med..

[42] Martino G.P., Benfaremo D., Bitti G., Valeri G., Postacchini L., Marchetti A., Angelici S., Moroncini G. (2022). 6 and 12 Month Outcomes in Patients Following COVID-19-Related Hospitalization: A Prospective Monocentric Study. Intern. Emerg. Med..

[43] Li D., Liao X., Ma Z., Zhang L., Dong J., Zheng G., Zi M., Peng W., Wei L., Li Z. (2022). Clinical Status of Patients 1 Year after Hospital Discharge Following Recovery from COVID-19: A Prospective Cohort Study. Ann. Intensive Care.

[44] Liao T., Meng D., Xiong L., Wu S., Yang L., Wang S., Zhou M., He X., Cao X., Xiong H. (2022). Long-Term Effects of COVID-19 on Health Care Workers 1-Year Post-Discharge in Wuhan. Infect. Dis. Ther..

[45] Zhao Y., Yang C., An X., Xiong Y., Shang Y., He J., Qiu Y., Zhang N., Huang L., Jia J. (2021). Follow-up Study on COVID-19 Survivors One Year after Discharge from Hospital. Int. J. Infect. Dis..

[46] Han X., Chen L., Fan Y., Alwalid O., Jia X., Zheng Y., Liu J., Li Y., Cao Y., Gu J. (2023). Longitudinal Assessment of Chest CT Findings and Pulmonary Function after COVID-19 Infection. Radiology.

[47] Bazdar S., Kwee A.K.A.L., Houweling L., De Wit-van Wijck Y., Mohamed Hoesein F.A.A., Downward G.S., Nossent E.J., Maitland-van Der Zee A.H. (2023). A Systematic Review of Chest Imaging Findings in Long COVID Patients. JPM.

[48] Fabbri L., Moss S., Khan F.A., Chi W., Xia J., Robinson K., Smyth A.R., Jenkins G., Stewart I. (2023). Parenchymal Lung Abnormalities Following Hospitalisation for COVID-19 and Viral Pneumonitis: A Systematic Review and Meta-analysis. Thorax.

[49] Bocchino M., Rea G., Capitelli L., Lieto R., Bruzzese D. (2023). Chest CT Lung Abnormalities 1 Year after COVID-19: A Systematic Review and Meta-Analysis. Radiology.

[50] WHO (2023). Clinical Management of COVID-19: Living Guideline, 18 August 2023.

[51] Yamamoto N., Bauer G. (2020). Apparent Difference in Fatalities between Central Europe and East Asia Due to SARS-COV-2 and COVID-19: Four Hypotheses for Possible Explanation. Med. Hypotheses.

